# Myths and realities: effort and response distortion in low-stakes, self-report assessments of noncognitive skills

**DOI:** 10.3389/fpsyg.2026.1785648

**Published:** 2026-05-04

**Authors:** Ross Markle, Zhen Wang

**Affiliations:** 1 ETS, Princeton, NJ, United States; 2 DIA Higher Education Collaborators, Nashville, TN, United States

**Keywords:** Likert scale, non-cognitive assessment, response distortion, response variance, validity

## Abstract

Over the past several decades, the higher education community has become increasingly interested in assessing noncognitive factors. Most tools still rely on self-report (e.g., Likert-type) items to measure these skills and behaviors, yet there are many concerns that arise from anecdotal or hypothetical behaviors—patterns of student responding to surveys that may threaten the validity of the results. This paper uses data from a national study of college students’ noncognitive skills to examine the extent to which two of these phenomena–low effort and response bias–occur. Although some students fit a profile that suggests potential inaccuracies, data show that such occurrences are rare and these hypothetical behaviors in low-stakes, self-report assessment pose a minimal threat to the validity of their use.

## Introduction

Low-stakes self-report assessments can help colleges and universities gather valuable information about the noncognitive skills and behaviors that play a crucial role in student success (Poropat, 2009; Richardson et al., 2012; Robbins et al., 2004). Non-cognitive skills (also often referred to as socioemotional skills) cover a range of abilities such as commitment, sensitivity to stress, academic self-efficacy and teamwork. These skills are critically important to student success in school. However, some practitioners avoid the use of potentially valuable assessment tools because they believe certain student behaviors – namely low effort toward the assessment and response distortion – make these tools invalid. Even those who use such measures may question the extent to which results are accurate.

In this study we used data from a large, multi-institutional national research study of noncognitive skills to examine the prevalence of several behavioral response profiles that might suggest threats to validity. The primary goals of this work are to (1) articulate some commonly perceived threats to validity; and (2) examine the extent to which those threats are present. Indeed, if low effort and response bias (a systematic error—a consistent over- or under-estimation of a score) are threats to validity, but if they are rare, they should not serve to deter institutions from using potentially valuable assessments. An additional goal is to (3) open the conversation about validity threats – particularly around tools that can help improve student success. Doing so can assuage concerns among practitioners while also encouraging other researchers to continue the conversation.

### The use and criticism of self-report measures

Self-report measures are the prevailing method of measuring noncognitive factors in both educational (Kyllonen et al., 2011; National Research Council, 2011, 2012; Strayhorn, 2006) and occupational (Spector, 1994; Paulhus and Vazire, 2007) settings. More specifically, researchers and practitioners typically employ what Pauhlus and Vazire refer to as an “indirect self-report.” Here, respondents are asked to respond to a series of questions or statements about their behaviors, attitudes, or perceptions, typically using yes-no, agree-disagree, “not at all like me” to “very much like me,” or similar type of response scale. Responses are then scored and aggregated to make inferences about latent constructs. The indirect self-report is contrasted to a direct self-report, where respondents are presented with a single, direct question about their level of the latent construct. A direct self-report example would be commercially produced or standardized Tests, for example, nationally standardized multiple-choice tests are widely used and assist departments in determining programmatic strengths and weaknesses when compared to other programs and national data. In the current study, any references to “self-reports” or “self-report measures” refer to indirect self-report methodologies.

Certainly, this type of measurement holds several advantages. For one, researchers generally hold “an implicit assumption that an individual is uniquely positioned to report on his or her standing on statements about the constructs and may well be the best source for the information” (National Research Council, 2011, p. 71). Or, as Pauhlus and Vazire put it, “If you want to know what Waldo is like, why not just ask him?” (p. 224). Self-report measures have also repeatedly been shown to significantly and sizably predict both educational (Poropat, 2009; Richardson et al., 2012; Robbins et al., 2004) and occupational (Barrick and Mount, 1991; Ones et al., 2007) outcomes. An additional advantage is that self-report items are generally simple to write, require short response times, and their responses are amenable to many traditional psychometric analyses and models (see Paulhus and Vazire, 2007).

### Faking

However, nearly every researcher who discusses the use of self-report measures also discusses their drawbacks. Perhaps the most notable concern is the ability of responses to be “faked,” or intentionally distorted in order produce more favorable scores. Technically “faking” typically means you intentionally distorted your responses to look a particular way while “response bias” is a broad classification that includes both intentional and unintentional distortions of responses. Meta-analyses have shown that respondents can both over-report their attributes when instructed to do so (Viswesvaran and Ones, 1999; Alliger and Dwight, 2000) and under-report negative characteristics such as psychopathology (Baer et al., 1992).

Messick (1979) identified faking as one of the threats to noncognitive assessment in educational settings. More recently, others (e.g., Kyllonen et al., 2011; Ones et al., 1996) have noted faking as an impediment to the adoption of noncognitive assessment in various settings and contexts, though they refer mostly to admissions decisions, which are high stakes. Dilchert et al. (2006) conducted a review of the literature on faking, as well as its prevalence, impact, and potential remedies. However, it is important to note that most of this research deals with the use of noncognitive assessments in personnel selection settings, where examinees have greater incentive to distort their responses than some educational settings (e.g., diagnostic or formative assessment). As such, research into the prevalence of faking in these low-stakes noncognitive situations is still necessary.

### Self-deception

In addition to overt faking, a similar concern with self-reports is self-deception. Paulhus (1984) distinguished between impression management, which involves the intentional distortion of responses (and would include faking), and self-deception, which is an unintentional positive bias toward one’s own self or characteristics. In their review of self-report measures, Paulhus and Vazire (2007) review two key issues. One is socially desirable responding (SDR), which is the propensity to provide more favorable responses or to avoid less favorable responses. Pauhlus and Vazire discuss both conditional SDR (i.e., in specific situations) and stable SDR, which has received considerable support in the literature and is viewed more as a characteristic of the respondent rather than a specific, situational behavior. Additionally, they also discuss the issue of “constraints of self-knowledge” as a potential limitation of self-reports. This concept opposes their original question (“If you want to know what Waldo is like, why not just ask him?”) by noting that individuals may either ignore or be unaware of pertinent information about themselves (see also Dunning et al., 2004).

### Low effort

Interestingly, other concerns about the use of some assessments relate to a lack of effort put toward these assessments. In “low-stakes” assessments, students are asked to complete measures of various skills – both cognitive and noncognitive – for the purposes of assessing programs (e.g., general education, student affairs, majors). Whereas one would surmise that faking takes place when students are overly motivated, in this case there is evidence of the exact opposite phenomenon: students having no motivation to respond, which also threatens the validity of their responses. As Thelk et al. (2009) put it: “It is quite reasonable to speculate that in testing situations for which no personal consequences exist, low examinee motivation may result in test performances that underestimate true student ability” (p. 129).

Some researchers have used response time as an indicator of examinee motivation in low-stakes assessment settings. Related to research on “rapid guessing,” this work uses response-time effort (RTE), or the amount of time an examinee spends on an individual item, to determine if examinees continually respond in an amount of time that would not allow for a sufficient examination of the item (Wise and Kong, 2005). RTE has been explored and supported in several studies, including demonstrations of its efficacy in “motivation filtering,” where responses with low RTE are removed to improve score validity (e.g., Schnipke and Scrams, 1997; Swerdzewski et al., 2011). Generally, this research has been applied to low-stakes measures of cognitive ability or academic achievement. There has been little if any investigation of response time as an indicator of motivation in noncognitive assessments.

### Support for self-reports

Despite both logical concerns and empirical evidence that faking can impact the validity of self-reports, support for their use is still prominent. In one of their meta-analyses, which reviewed personality assessment in a clinical context, Costa and McCrae (1992) concluded that “there is substantial evidence that self-reports from patients are, in general, trustworthy” (p. 8). In that same study, they found notable agreement between self-reports and the reports of others in measuring an individual’s personality.

The key to the impact of faking on self-reports may rest in the use of assessment scores. In their book entitled *New Perspectives on Faking in Personality Assessment*, Ziegler et al. (2011) stated three conditions for faking: (1) the respondent perceives faking as necessary, (2) the respondent believes themselves capable of faking, and (3) the outcome [to be achieved by a positive score] has value to the respondent. To be sure, these conditions might easily exist in many potentially valuable uses of assessment, such as employment selection and undergraduate or graduate admissions. However, one could easily intuit that a student completing noncognitive battery for diagnostic or formative purposes – assuming they were formally and properly instructed of the assessments intended use – would fail to meet all or even any of these criteria.

The use of an assessment may also refute another criticism of self-reports: correlations between self-reports and reality. Dunning et al. (2004) reviewed several examples where self-reports had low or modest correlations with established criteria, including students’ perceived knowledge and test scores and doctors’ perceived knowledge and professional exams. Paulhus et al. (1998) found that, even with focused efforts on improved measurement, self-report and indirect measures of intelligence correlated no more than 0.30 with direct measures of IQ.

But the factual validity of an individual response may not always be the primary criterion of a self-report’s value. As economist Friedman (1953) once said:

*… a theory cannot be tested by comparing its ‘assumptions’ directly with ‘reality.’ … the question whether a theory is realistic ‘enough’ can be settled only by seeing whether it yields predictions that are sufficiently accurate for the purpose in hand. (p. 15)*

Consider the University Attachment Scale put forth by France and Finney (2009). The authors constructed a 9-item, self-report measure designed to address college students’ perceived connection to both the people within an institution (member attachment) and the institution as a whole (group attachment).

One of the items asked respondents to rate to the extent which they were like the typical student at that institution. Certainly, one could evaluate this item in terms of its factual validity (do students who agree with this item represent the demographic, academic, or personality make-up of most other students at their institution?). But for the use of this measure – to identify students’ perceived attachment to the university and its members – the greater validity issue centers around students perceived connections, how that relates to the construct, and how that will indicate a student’s future engagement with the university. Regardless of how like the “typical student” a respondent might be, the authors hypothesized that these connections were key to students’ success. Indeed, France, Finney, and Swerdzewski found that students who were involved in co-curricular activities had higher attachment scores than noninvolved students, and transfer students had lower scores than nontransfer students.

Similarly, many of the uses of noncognitive assessments in higher education are not concerned with the factual validity of self-reports. Assessments might be used to place students into college level courses (Boylan, 2009; Burdman, 2012; Conley, 2007); predict student grades and persistence (Poropat, 2009; Richardson et al., 2012; Robbins et al., 2004), or to understand how students access resources on campus (Robbins et al., 2009). Certainly, in each of these cases, factual validity is an important consideration of assessment use, but a lack of complete factual validity could be tolerated if assessment results lead to better understanding of students, guidance in working with them, and ultimately improvements in student success.

### Methods of detection

In addition to RTE, Previous research has used several tools to examine issues of effort and response bias in noncognitive assessment. These include the use of dedicated (and often embedded) measures within the assessment, such as measures of socially desirable responding (e.g., Paulhus, 1988), measures of reported effort toward and perceived importance of a given assessment (Thelk et al., 2009), and scales designed to detect patterns of potentially invalid responses (i.e., “validity scales,” see Piedmont et al., 2000).

These scales have some desirable properties, namely that they have a direct, quantifiable, and individual measure of the construct that is tied to the underlying threat to validity. However, they still rely on the reports of the examinee, and are thus still susceptible to both response bias and lack of effort. What’s more, as discussed, some of the constructs may reflect personality traits of the individual, rather than contextual response sets (see Paulhus and Vazire, 2007).

Other tools, such RTE, use observations of examinees’ behavior to make inferences about their performance. Because these methods are not reliant on responses, they avoid several of the challenges tied to dedicated measures. One setback might be the inferential leap drawn from the observation of behaviors. For example, with RTE, it is possible that students might read items very quickly, and not be exerting low effort.

### The current study

Amid these concerns and questions about the use of self-report measures of noncognitive skills, research is needed to examine whether or not these threats to validity that have been identified in other contexts take place in higher education settings, particularly given the different uses of assessment results. If so, to what extent does that happen? Is there existence sufficiently prevalent to impede the use of these assessments?

In the current study, we used data from a national study of student success in higher education to examine these questions. Given this use of these archival data, intrusive means of examining the impact of faking behavior were not available (e.g., dedicated scales). However, by using data from this observational setting, we were able to observe the extent to which several behavioral profiles were present. Although these profiles were not perfect indicators of a lack of effort, response bias, or faking, they are used here to flag observations in which these behaviors were more likely to be present.

## Methods

### Participants and administration

Data were taken from a national study of college student success that included 4,729 students from 10 colleges and universities. Students completed a battery of noncognitive skills early in their academic careers, in settings such as new student orientation, student success courses, placement testing sessions, and entry-level math and English courses. In addition to the venue, other specifics of sampling and administration were under the control of participating institutions, and thus varied somewhat across institutions. For example, while the assessment was always administered online, students could be complete the assessment at home or on-campus, depending on the institutional administration.

Overall, and given the previous discussion of assessment use, it is important to note that students took this assessment in very low-stakes conditions. Regardless of the administration venue, students were given instructions that this assessment was diagnostic and would only be used for institutional research purposes. Although institutions were permitted to vary in the instructions given to students, an example introductionused with one school’s orientation program is provided below:

*“... we would like to gather some further information about you. Below is a link to an assessment that will ask you several things, including your attitudes toward [the institution], the way you study and approach class, and some other characteristics about yourself. This is a research project that is being conducted in order to better understand your needs and learning style as a student and how [the institution] can make your undergraduate learning experience more enriching. This information is very important to help us understand incoming students and helps us a great deal in developing future Orientation experiences... Note, the information you provide is entirely confidential, and your individual responses will not be viewed by anyone on campus.”*

Although this does not represent some of the individual uses of noncognitive assessment (e.g., course placement, advising) that might take place in larger contexts, these instructions were designed to help students understand that individual, high-stakes decisions would not be made. Or, in reference to Ziegler et al. (2011) conditions for faking, these instructions were designed to inform students that faking was not necessary.

### Data sources and materials

*SuccessNavigator*™ assessment, a broad-based psychosocial assessment designed to improve success by indicating a given student’s likelihood for academic and retention success, as well as providing developmental feedback and tailored support strategies. A battery of 10 noncognitive skills was used for this study (see Table 1), measuring factors such as organizational skills, class engagement, commitment to college goals, responses to stress, academic self-efficacy, and social connections (see Markle et al., 2013, for information on scale development and validity evidence). Total testing time was measured automatically by the online assessment platform. Because we were interested in behavior across the entire assessment session, response variance was calculated across the full battery of 183 items, though the final noncognitive scales (after factor analyses were used to evaluate scale structure and remove poorly fitting items; Markle et al., 2013) ranged from 7 to 11 items in length and used a total of 93 items.

Each item used a 6-point, Likert-type response scale using “strongly disagree” to “strongly agree.” Demographic and background information and academic achievement factors (e.g., standardized admissions and placement test scores and high school GPA) were taken both from student self-reports and institutional data provided by the schools. When both sources of data were available, institutional records were preferred. The *SuccessNavigator* assessment is geared toward the complete span of students entering two- and four-year public and private postsecondary institutions. This includes full- and part-time students: students entering upon high school graduation, residential and commuter students, older students returning to campus, military veterans, first-generation college goers, and workers in transition.

**Table 1:** Noncognitive scales, number of items, and reliability (Markle et al., 2013).

Noncognitive factor	Definition	Items	Alpha
Meeting class expectations	Doing what’s expected to meet the requirements of your course including assignments and in-class behaviors.	10	0.83
Organization	Strategies for organizing work and time.	9	0.80
Commitment to college	Perceived value and determination to succeed in and complete college.	9	0.84
Institutional commitment	Attachment to and positive evaluations of the school.	8	0.90
Sensitivity to stress*	Tendency to feel frustrated, discouraged, or upset when under pressure or burdened by academic demands.	10	0.88
Test anxiety*	General reactions to test-taking experiences, including negative thoughts and feelings (e.g., worry, dread).	9	0.88
Academic self-efficacy	Belief in one’s ability to perform and achieve in an academic setting.	9	0.86
Connectedness	A general sense of belonging and engagement.	7	0.86
Institutional support	Attitudes about and tendency to seek help from established resources.	11	0.86
Barriers to success*	Financial pressures, family responsibilities, conflicting work schedules, and limited institutional knowledge.	11	0.78

*For negatively labeled scales, items were scored such that higher levels of the scale refer to more adaptive strategies. For example, “higher” test anxiety scores mean lower levels of anxiety in testing situations.

The *SuccessNavigator* assessment produces several key pieces of information to help students, faculty, staff, and the institution as a whole. First, psychosocial factors are outlined using four broad areas – *Academic Skills*, *Commitment*, *Self-Management*, and *Social Support* – which can be used to understand the factors that might facilitate or hinder a student’s success. Each of these four areas also includes subscores that further define strengths and weaknesses. The *SuccessNavigator* assessment scores have been developed and supported by a rational-empirical approach that uses both established theory and psychometric evidence (see Markle et al., 2013). Additionally, the *SuccessNavigator* assessment captures students’ admissions or placement test scores and high school GPA, when available. These data can be uploaded by the institution or reported by the student. Capturing these data supports a holistic understanding of students that includes both cognitive and noncognitive indicators.

Student academic test scores, high school GPA, and the *SuccessNavigator* assessment scores are then combined to indicate how well students are likely to do in their first year of postsecondary education. One score—the *Academic Success Index*—is tied to a student’s GPA, with higher scores indicating a higher first-semester GPA. Another index—the *Retention Success Index*—is tied to persistence to the second semester, with higher scores indicating a greater likelihood of returning.

### Data analysis

#### Response time

The analyses took place in two stages. In stage one, we examined the prevalence of two kinds of low-engagement response sets. One of these deals with rapid responding, where students completed the assessment in less than 15 min. Initial pilot testing provided an estimated response time of roughly 30 min, thus one half of that time was used as an initial criterion for rapid responding.

#### Response variance

We also examined responses and the variance of responses across items. There are several ways in which response variance might relate to the validity of responses. Generally, across a litany of statements, one could intuit that a student might use the entirety of a response scale, tending to agree with some items and disagreeing with others. This is particularly true in the present study, where 92 of the 183 items were reverse-coded. However, a student who was not engaged with the assessment, such as one who was inattentively and repeatedly clicking the same response, would have lower response variance.

One challenge to using response variance in this setting is the availability of metrics. Lacking statistical tests or metrics that could indicate a sufficiently variant set of responses, we created several benchmarks designed to represent what different levels of response variance might look like. Obviously, a zero variance response set would use the same scale point (again, ranging from 1 to 6) for all responses (*σ^2^* = 0). Admittedly, this scenario is highly unlikely given the number of items and the percentage of negatively worded items. The opposite variance scenario would be maximal variance – an equal number of 1’s and 6’s in the response set (*σ^2^* = 6.28). The variance scenarios in between are admittedly arbitrary, but simply designed to demonstrate an example response set at that level of variance.

**Figure 1:**
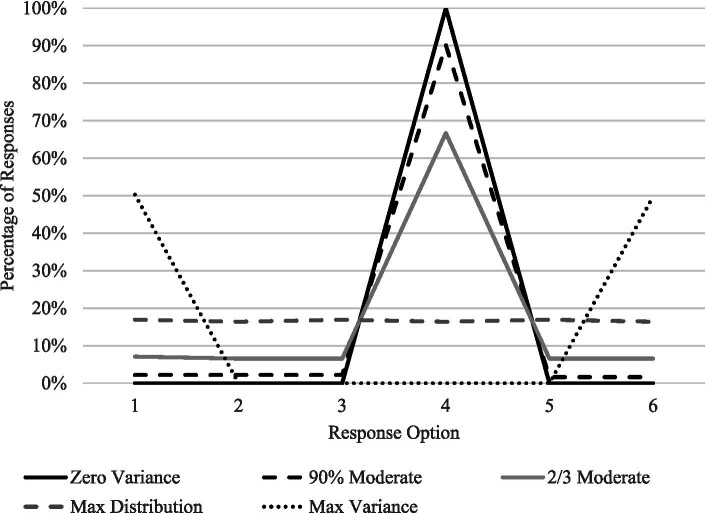
Distribution of responses on a six-point scale for example variance scenarios.

Figure 1 shows the distribution of responses for the response sets that indicate the selected variance scenarios. The aforementioned maximal variance scenario, as well as the zero variance scenario (with 100% of responses selecting 4 on the scale) are shown. A maximal distribution variance scenario – where a respondent uses each point on the response scale equally – is also shown. Two other points – the “90% moderate” and “2/3 moderate” - show greater tendency toward selecting one response (4 was chosen in this case). In each of these cases, all of the minority responses (i.e., not equal to 4) were randomly distributed among other response categories.

**Figure 2:**
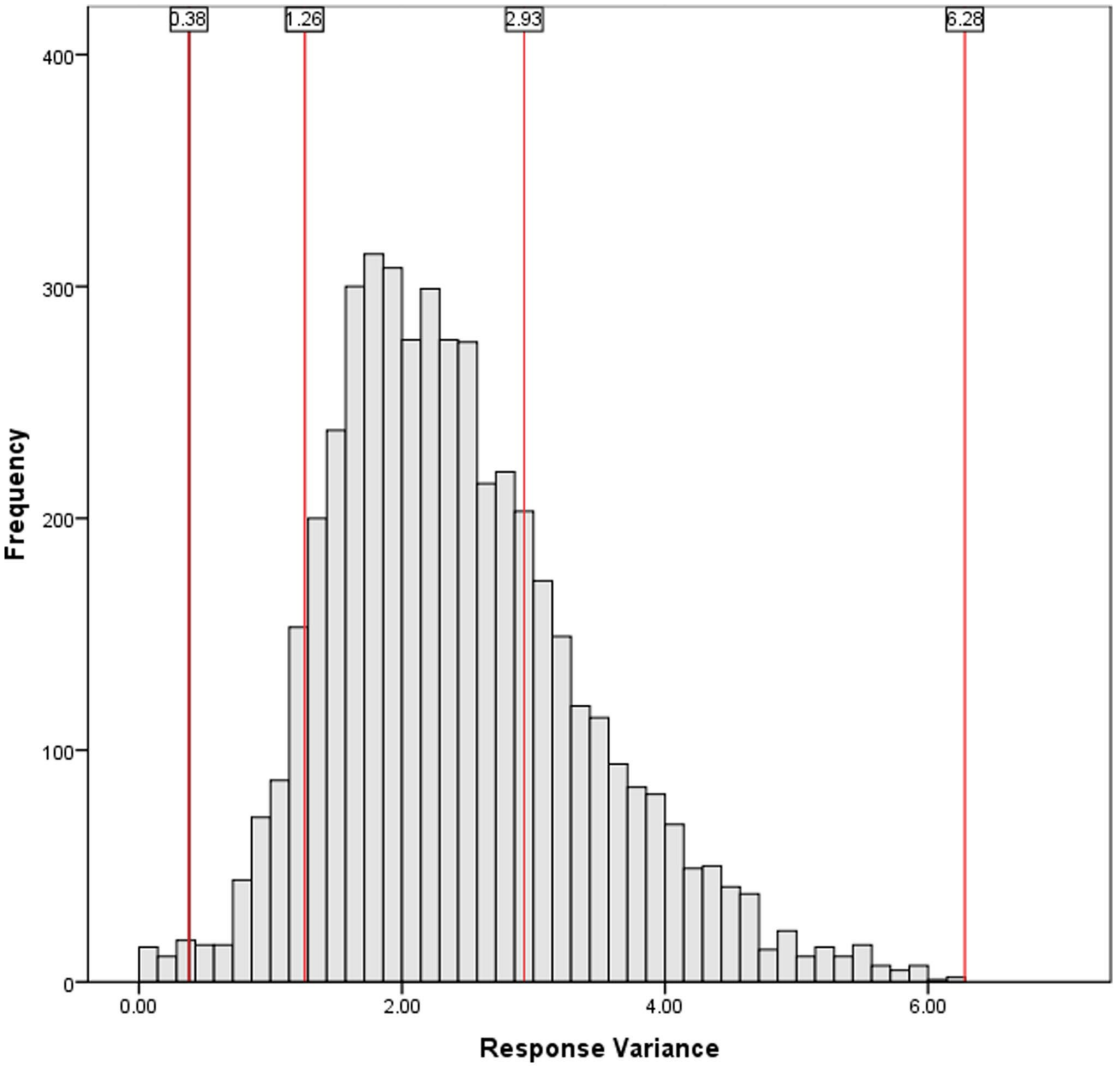
Frequency distribution of response variances, including benchmarks at various demonstrative response sets.

When applying these profiles to the same assessment setting as the participants of this study, variance benchmarks can be created. Again, the zero variance profile, were *σ^2^* = 0, is unlikely, though useful for demonstration in Figure 1. The 90% moderate response profile – whereby 90% of responses were the fourth scale point and 90% of responses were randomly distributed across other categories – yielded *σ^2^* = 1.26. For the 2/3 moderate response profile, variance was significantly higher (*σ^2^* = 2.93). Finally, the maximum response variance possible (i.e., an equal number of minimum and maximum score points selected) yielded the highest variance (*σ^2^* = 6.28), though this benchmark is essentially unrealistic in practice. These benchmarks are demonstrated in juxtaposition to actual response data in Figure 2.

Once again, these scenarios are not intrinsically meaningful. Moreover, there are other ways these response sets could have been created (e.g., using a different scale point for the zero variance and majority selections, not evenly distributing non-majority responses across scale points). But, yet again, the goal of this exercise was not to present meaning criteria for response variance, only to demonstrate what one response set might look like at a given level of variance.

### Potentially distorted responses sets

The second stage of the analyses examined potentially inaccurate responses due to response distortion, which is usually an indication about changes in relationships. One perception of low-stakes, self-report items is that students will simply provide the desired response, either through intentional deception or over-estimation of their own skills and behaviors. In the current assessment environment, each score is broken into three categories based on the normative distribution of scores: low (bottom 25%), medium (middle 50%), and high (top 25%). Thus, we examined the percentage of scores in which students demonstrated high scores across all 10 scales (see Table 1) as one possible example of socially desirable responding.

In each of these cases – low response time, low response variance, and high noncognitive scores – the behavioral profile does not guarantee that the student has provided an invalid response. For example, students with strong reading ability could very well respond considerably faster than students with reading challenges. Additionally, students could legitimately have very strong noncognitive skills. In fact, given that high scores, in the present assessment, are based on normative distributions, profiles of all high scores are likely to occur in some cases. Low response variance may be attributable to item characteristics rather than response behaviors.

Overall, while making student-level inferences about the validity of an individual’s response may be limited, these behavioral profiles simply represent examples of situations in which students may be more likely to have distorted their responses. Below, results are organized by these three potential characteristics of responses –response time (effort), response variance (effort), and score variance (distortion) – which address different aspects of effort and response distortion.

## Results

### Response time (effort)

Our results suggested that very few students engaged in rapid responding behavior. Only 0.30% (*n* = 14) of students completed the assessment in 15 min or less. Even expanding the criterion for rapid responding to 20 min (*n* = 112; 2.37% of responses) suggested very low rates of this behavior. There are two caveats to this finding. Even though response rates for several participating institutions were high – in many cases exceeding 50% of students invited to participate – participation was usually voluntary. Thus, those students who wished not to participate could have simply not responded to the assessment and, in a less voluntary administration setting, may have rapidly responded. Second, response time was captured very simply according to the times a student began and completed the assessment.

### Response variance (effort)

The next step in examining low-engagement response styles was to look at respondents with low variance in their responses. Admittedly, developing an operational definition of “low variance responding” was difficult. As expected, very few students exhibited a zero response variance set (*n* = 4; < 0.01%). Figure 2 shows the distribution of response variances, and includes benchmarks at the 90% moderate (i.e., 90% of responses were a single chosen value of 4, the remaining responses evenly distributed); 2/3 moderate (i.e., 2/3 of responses choosing value of 4, remaining responses evenly distributed), maximal distribution (i.e., responses evenly distributed across all options), and maximal variance (i.e., responses evenly distributed between 1 and 6).

The results suggested that low-variance responding was not notably present in the current sample. Very few students (*n* = 39; 0.82%) had a response variance lower than the 0.38 benchmark, which is the variance of the 90% moderate response set. The vast majority of students (*n* = 4,333; 91.6%) had a higher response variance than the 2/3 moderate benchmark, and a notable number of students (*n* = 1,265; 26.7%) met or exceeded the variance threshold shown by the maximal distribution profile (i.e., selecting all responses equally), suggesting they used a wide array of responses across the scale.

### Score variance (distortion)

Finally, we examined behavioral profiles that may contain a greater likelihood for distorted responses, either through intentional impression management or through overestimation of one’s own skills and behaviors, as indicated by the number of high scores found within students’ reports. Overall, we found a low likelihood of distorted profiles, with only 47 students (0.99%) receiving high scores in all 10 noncognitive areas and only 296 students (6.26%) receiving high scores in 8 or more areas. Compared to over-reporting positive characteristics, there may, however, be some evidence that students under-report negative skills and behaviors. Nearly one quarter of students (*n* = 1,168; 24.7%) of students had zero low scores in their profile.

It should be noted that, given the normative way in which high (top quartile), medium (quartiles two and three), and low scores (bottom quartile) are determined, there are statistically expected base rates for each of these profiles. For example, the probability of a student receiving all high scores can be calculated by taking 0.25^10^ (a 0.25 probability of receiving a high score across all 10 scales), and would thus occur in less than 0.001% of cases (*n* = 0 given the current sample size). Conversely, the probability of receiving zero low scores is equal to 0.75^10^ (0.75 chance of receiving a medium or high score across all 10 scales), or 5.63% of cases (*n* = 266 in a sample of 4,729). This, of course, assumes that scores are independent, which is not the case, given established covariance among the scales (see Markle et al., 2013). Thus, these calculations represent underestimates of the actual base rates of the “all high” and “zero low” profiles.

## Discussion

### Limitations

Before discussing the implications of and future directions for this research, there are notable limitations to the current study. We have emphasized here that assessment use is a critical factor when considering the extent to which effort and response bias are threats to validity. In the current study, participants may have viewed the assessment as a research study with essentially no individual relevance. However, there are other settings in higher education in which the use of self-report, noncognitive measures has potential use. In some of these (e.g., admissions), the susceptibility of these measures to faking and coaching might create obvious threats to validity.

In other settings, such as using noncognitive measures for diagnostic feedback, advising, and course placement (e.g., Markle et al., 2013), the generalizability of these findings is unknown. In situations where students receive individual feedback or score reports, there is likely to be decreased instances of suboptimal effort, but there may also be increased internal motivation to over-state one’s skills and behaviors. Future research should certainly explore the instances of these and other behavioral profiles in other settings.

The current findings are also limited in the inferences that can be drawn from the current behavioral profiles. As mentioned, shorter response times may be indicative of a lack of effort, but might also be found when students with strong reading skills complete the assessment quickly. Low response variances may be due to a lack of engagement and limited use of the response scale, but they might also be legitimate response patterns. Conversely, high response variances may be attributable to the high number of reverse-scored items, and might be susceptible to other type of response bias (such as acquiescence; see Knowles and Condon, 1999, as an example). Lastly, students with large numbers of high scores might actually possess those profiles of skills. Once again, each of these response behaviors is not wholly indicative of a threat to validity, they simply represent cases in which an invalid response is somewhat more likely.

### Implications

Overall, there are two important contributions of this study to the larger field of diagnostic noncognitive assessment in higher education. First, these findings attach actual data to some of the anecdotes of low-stakes, self-report assessments. Understandably, some current and potential users of these assessments fear that students will not take these measures seriously, or may not provide accurate reports of their own skills and behaviors. Nevertheless, the data show that students rarely responded quickly or with low variance, nor was there a notable proportion of excessively high scores. Though there is some evidence that students may be more likely to under-report negative characteristics, this finding should be further explored. One practical implication for users of an assessment such as this is that students with moderate scores may indeed have greater challenges, and might require more significant intervention in these noncognitive areas.

The second contribution of this study was to demonstrate various behavioral profiles that might be used to observe low effort or response bias. Although there is a sizable extant literature on faking in high takes (namely personnel selection) settings, there has been less investigation into the prevalence and impact of response distortion in low stakes settings. Given the available data, we were able to identify three characteristics of responses – response time, response variance, and score distribution – though others are certainly available. Given the limitations of dedicated or embedded measures of response distortion, scales to measure effort, socially desirable responding, or other response characteristics might not always be feasible. Moreover, for the goal of this study – to identify the prevalence of behaviors across a population and not necessarily detect individual characteristics – behavioral measures can be used without consuming assessment time and effort.

## Future directions

Although the concepts and behavioral profiles put forth in this study provide an example, future research, learning from both the response time research in cognitive ability testing and the noncognitive faking research conducted in personnel settings (e.g., Dilchert et al., 2006), should seek to more directly and effectively identify these behavioral sets that might indicate invalid responses in low stakes settings. For example, using item-level response time or response variance across smaller sets of items might help to identify lack of effort or response bias at specific points in the assessment, rather than across an entire assessment experience. For example, item-level data might identify students who were initially motivated to participate, but then disengaged later and began responding rapidly.

Aside from the existence and prevalence of effort and response distortion, another key issue deals with the impact of these phenomena. Earlier, we discussed the issue of factual validity and its potential irrelevance to the validity of inferences drawn from such assessments in higher education, particularly in diagnostic or predictive terms. When considering these responses alongside other outcomes (e.g., retention, GPA, graduation), future research can determine if distorted responses – either within individuals or across groups (e.g., demographic groups) – have a notable impact on the validity of certain inferences.

While respondents’ time and variance might provide insight into a lack of effort, response distortion is an issue that continually affects (or is at least associated with) personality assessment. Thus, new methodologies have been developed to fundamentally address these issues. The use of forced-choice assessments – where respondents must choose between two or more equally desirable statements, rather than agreeing with singular statements – has been explored in personnel settings as a means of reducing response distortion (Christiansen et al., 2005; Heggestad et al., 2006; Jackson et al., 2000), and might provide a remedy in educational settings as well.

## Conclusion

At present, the majority of noncognitive assessment in higher education relies on students’ self-reports. Given persistent questions about students’ effort and response bias on such measures, it is important to understand if these behaviors exist and to what extent. The current study provides an initial glimpse into these behaviors in low-stakes settings, and shows that their occurrence appears to be rather limited.

## Data Availability

The raw data supporting the conclusions of this article will be made available by the authors, without undue reservation.
